# Increased serum levels of tumour-associated trypsin inhibitor independently predict a poor prognosis in colorectal cancer patients

**DOI:** 10.1186/1471-2407-10-498

**Published:** 2010-09-17

**Authors:** Alexander Gaber, Björn Nodin, Kristina Hotakainen, Elise Nilsson, Ulf-Håkan Stenman, Anders Bjartell, Helgi Birgisson, Karin Jirström

**Affiliations:** 1 Center for Molecular Pathology, Department of Laboratory Medicine, Lund University, Skåne University Hospital, Malmö, Sweden; 2 Department of Clinical Chemistry, University of Helsinki and Helsinki University Central Hospital, Helsinki, Finland; 3 Department of Clinical Sciences, Division of Urological Cancers, Lund University, Skåne University Hospital, Malmö, Sweden; 4 Department of Surgery, University of Uppsala, Uppsala, Sweden

## Abstract

**Background:**

There is an insufficient number of reliable prognostic and response predictive biomarkers in colorectal cancer (CRC) management. In a previous study, we found that high tumour tissue expression of tumour-associated trypsin inhibitor (TATI) correlated with liver metastasis and an impaired prognosis in CRC. The aim of this study was to investigate the prognostic validity of serum TATI (s-TATI) in CRC. We further assessed the prognostic value of carcino-embryonic antigen in serum (s-CEA) and the interrelationship between s-TATI and TATI in tissue (t-TATI).

**Methods:**

Using an immunofluorometric assay, s-TATI levels were analysed in 334 preoperatively collected serum samples from patients with CRC. Spearman's Rho and Chi-square test were used for analysis of correlations between s-TATI and clinicopathological parameters, s-CEA and t-TATI. Kaplan-Meier analysis and Cox uni- and multivariate regression analysis were used to estimate disease free survival (DFS) and overall survival (OS) according to quartiles of s-TATI and cut-offs derived from ROC-analysis of s-TATI and s-CEA.

**Results:**

Increased levels of s-TATI were associated with a reduced DFS (HR = 2.00; 95% CI 1.40-2.84, *P *< 0.001) and OS (HR = 2.40; 95% CI 1.74-3.33, *P *< 0.001). (HR = 2.89; 95% CI 1.96-4.25). This association remained significant in multivariate analysis. The association for OS remained significant in multivariate analysis (HR = 1.51; 95% CI 1.03-2.22, *P *= 0.034 for DFS and HR = 1.78; 95% CI 1.25-2.53, *P *= 0.001 for OS). There was no significant association between s-TATI and t-TATI. The prognostic value of s-CEA was also evident, but somewhat weaker than for s-TATI.

**Conclusions:**

High preoperative s-TATI levels predict a poor prognosis in patients with CRC, and the prognostic value is independent of established prognostic parameters and t-TATI expression. These data suggest that s-TATI might be a useful marker for prognostic stratification in CRC.

## Background

Colorectal cancer (CRC) is one of the most common forms of cancer worldwide with approximately 1 million new cases detected every year [1]. Early detection, adequate surgical excision and optimal adjuvant treatment are of critical importance for outcome. Although several prognostic and predictive CRC biomarkers have been proposed [2], serum carcino-embryonic antigen (s-CEA) is currently the only accepted marker incorporated into clinical practice. S-CEA is used for early detection of metastasis during follow-up of patients with stage II and III disease and for monitoring response to adjuvant treatment in stage IV disease.

In a previous study by our group, we found that a high expression of tumour-associated trypsin inhibitor (TATI; synonymous to pancreatic secretory trypsin inhibitor, PSTI, and serine protease inhibitor Kazal type 1, SPINK1) in tumour tissue (t-TATI) was associated with an increased risk of metachronous liver metastasis and an impaired prognosis in CRC patients [3]. These findings are supported by *in vitro *data, demonstrating that TATI promotes invasiveness of CRC cells [4].

Several studies have found s-TATI to be of potential prognostic value in ovarian cancer [5,6], a good serum marker for monitoring [7] and prognosis [8] of bladder cancer, prognosis of renal cancer [9] and more accurate than CEA, carbohydrate antigen (CA) 15-3, CA 125 and CA 19-9 in post-operative follow up of renal cancer patients [10]. Previous studies on s-TATI in various cancer forms have been performed on rather small cohorts with diverging conclusions regarding its prognostic value. In a study from 1991, s-TATI was found to be a good predictor of liver metastasis in CRC and breast cancer [11]. Satake *et al *found elevated s-TATI concentrations in CRC patients, but the results were not considered to be of sufficient diagnostic value for clinical use [12]. In a study on 62 CRC patients, Pasanen *et al *found s-TATI to be a useful biomarker for staging of CRC, however less useful than s-CEA [13]. Similar results were obtained in another study comprising 53 CRC patients [14]. Solakidi *et al *found s-TATI to be a useful complementary biomarker for diagnosing and monitoring of gastrointestinal malignancies, having a higher sensitivity than s-CEA [15].

Three main mechanisms have been proposed to cause increased levels of TATI in serum; leakage from tumour-derived TATI into the circulation and as a response to tissue destruction and inflammation [16]. A transitory elevation of s-TATI levels have been found after surgery, suggesting that TATI may behave as an acute phase protein [14,17]. Elevated levels of s-TATI can also be detected temporarily in some non-malignant conditions, especially in pancreatitis [18], and in severe inflammatory diseases, injuries, and sepsis [12,19].

The purpose of the present study was to examine the prognostic value of s-TATI in a cohort of 324 prospectively collected CRC patients, including 308 cases previously analysed for t-TATI [3]. Furthermore, the prognostic value of s-CEA was assessed, as well as the association between s-TATI and t-TATI.

## Methods

### Patients

The original cohort consisted of 337 prospectively collected patients undergoing surgery for CRC at the Central District Hospital in Västerås, Sweden, between June 2000 and December 2003. Tumour tissue for construction of tissue microarrays (TMA) was available from 320 (95%) patients [3]. Pretreatment serum samples were available from 325 patients and s-TATI could be analysed in 324 (96%). Both tissue and serum data were available from 308 patients (91%), Serum data was available for 275 (82%) curatively treated patients.

Median follow-up time for surviving patients with samples available for s-TATI analysis was 6 (range 4-8) years. Recurrent disease was reported in 54 (19%) of curatively treated patients while 119 (37%) patients died during the study period. Preoperative radiotherapy (RT) was given to 84/108 patients with rectal cancer. All patients under 75 years of age with stage III colon cancer (*n *= 36) and 22 of 29 rectal cancer patients received adjuvant chemotherapy as well as some patients with high risk (T4, low differentiation) stage II disease (13/71). Palliative chemotherapy was given to 23 of 27 patients <75 years with stage IV disease.

Ethical approval for the study was obtained from the Ethic's committee at Uppsala University (ref no 00-001), whereby all patients gave their informed consent for participation in the study.

### Immunofluorometric assay of s-TATI

Serum samples were drawn prior to surgical treatment and stored at -70°C until analysis. The samples were analysed using a time-resolved immunofluorometric assay [20]. MAb 6E8 was used as a capture antibody for TATI and a europium (Eu) labelled antibody 11B3 was used as a tracer. Fluorescence was measured with a 1234 Victor 2 time-resolved fluorometer (Wallac, Turku, Finland. The lower limit of detection for TATI is 0.1 μg/l and the measuring range 0.5-90 μg/l.

### S-CEA

CEA in serum was determined by ELISA with commercially available MTPL-EIA kit (RE59101, IBL IMMUNO BIOLOGICAL LABORATORIES; http://www.ibl-hamburg.com). According to the manufacturer's instructions, the assay has a detection limit of 1 μg/l and a measuring range of 5-75 μg/l.

### Statistics

Correlations between s-TATI and clinicopathological characteristics were calculated using Spearman's Rho-test and Chi-square test. Kaplan-Meier analysis and log-rank test were used to estimate the relationship between different strata of s-TATI levels, disease free survival (DFS) and overall survival (OS). Endpoints for DFS and OS were defined as recommended by Punt *et al *[20]. Curatively treated patients were defined as patients with Stage I-III disease and microscopically free resection margins (R0). All observations were censored at the end of the study period (15^th ^November 2008). The Kolmogorov-Smirnov test was used to verify a normal or skewed distribution. Bivariate correlation analysis between s-TATI and s-CEA was performed. Optimal cut-off values were determined using a receiver operating characteristic (ROC) analysis including all patients in relation to OS. The TATI concentration at the ROC curve's closest point to the top of Y-axis (1.0) was used as cut-off. Quartile-concentration thresholds for the whole group were used for comparison. A Cox proportional hazards model was used for estimation of hazard ratio (HR) in both uni- and multivariate analysis after adjustment for age, gender, disease stage, differentiation grade, lymphatic or vascular invasion, s-CEA, s-TATI and t-TATI. Statistical analyses were carried out using the Statistical Package for Social Sciences, SPSS 16.0 package (SPSS Inc, Chicago, Ill) while ROC curve analysis and comparison of ROC curves and optimal cut-off calculation was performed using MedCalc statistical software 11.2 (MedCalc Inc., Mariakerke, Belgium). A *P*-value less than 0.05 was considered significant.

## Results

### Distribution of TATI in serum

**Figure 1:**
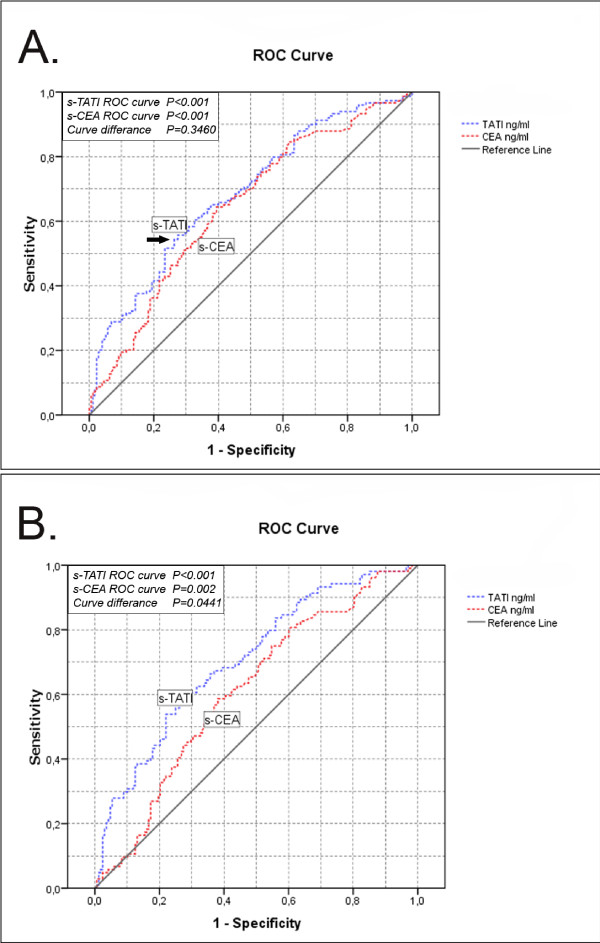
**ROC curve analysis**. ROC curve analysis calculated with the use of survival data (OS), showing AUC on s-TATI compared to s-CEA in all patients (A), curatively treated patients (B). Cut-off based on the point farthest to the northwest (black arrow) extracted from ROC curves data table (data table not shown).

The range of s-TATI concentrations in CRC patients was 4.4 μg/l to 169 μg/l (mean; 23.5 μg/l, median; 13.5 μg/l). The distribution was skewed and this was verified by Kolmogorov-Smirnov's test (*P *< 0.001). Based on ROC analysis the optimal cut-off was set to 15.59 μg/l. The AUC value for prediction of OS (Figure 1A) was 0.684 for s-TATI (*P *< 0.001) and 0.644 for s-CEA (*P *< 0.001). In curatively treated patients the AUC (Figure 1B) for s-TATI was 0.706 (*P *< 0.001) and 0.612 for s-CEA (*P *= 0.002) (Figure 1B). The range of the s-TATI quartiles were 4.346-10.432, 10.433-13.544, 13.545-22.039, 22.040-168.570 μg/l.

### Correlation between s-TATI and t-TATI

There was no significant association between s-TATI and t-TATI (continuous percentage of positive cells) (Spearman's Rho = 0.031, *P *= 0.591). This was also evident in separate analysis of curatively treated patients, different disease stages as well as colonic versus rectal cancer (data not shown).

### Correlations between s-TATI and clinicopathological characteristics

**Figure 2:**
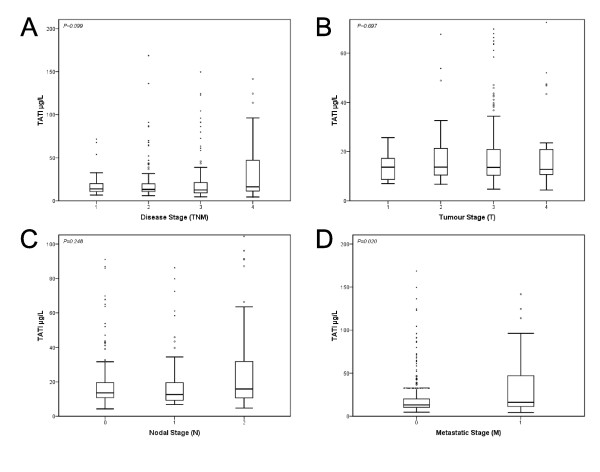
**Distribution of s-TATI according to disease stage**. Box plots displaying s-TATI distribution in TNM Stage and subcategories. Boxes show 25^th^, 50^th, ^75^th ^percentiles, whiskers show 5^th ^and 95^th ^percentile. Outliers are represented with circles while black dots represent extreme outliers. Distribution of s-TATI according to TNM stage (A), tumour stage (B), nodal stage (C) and metastatic stage (D). Kruskal Wallis test was used for statistical analysis.

Higher s-TATI levels were significantly associated with M stage (*P *= 0.02), but not with TNM stage, lymph node (N) or tumour (T) stage (Figure 2). As shown in table 1, s-TATI was significantly associated with higher age at diagnosis (*P *< 0.001) and right-sided tumour location (*P *< 0.001). Patients with cancer in the colon had significantly higher s-TATI levels than rectal cancer patients (*P *= 0.001). Notably, preoperative RT had been given to 84/107 (79%) rectal cancer patients in the evaluated cohort and in patients who had received preoperative RT (n = 85, all but one with rectal cancer), s-TATI levels were significantly lower than in patients who had not (*P *= 0.001). There was no significant difference in s-TATI levels between left-sided colonic tumours and rectal cancers (data not shown). In contrast to s-TATI, s-CEA levels did not differ significantly in relation to tumour location or preoperative RT and neither did t-TATI (data not shown).

**Table 1:** Correlation between TATI serum levels and clinicopathological characteristics

Categories calculated via ROC-curve:	< 15.59	> 15.59	*total*	*P-value*	*missing*
**Ranges (s-TATI μg/l)**	4.346-15.587	15.588-168.570			
*n*(%)	192(59.3)	132(40.7)	*324(100)*		
					
**Age**					
< 75	129(69.7)	50(40.7)	*179(58.1)*		
≥75	56(30.3)	73(59.3)	*129(41.9)*	< 0.001**	16(4.9)
					
**Gender**					
Female	90(46.9)	71(53.8)	*161(49.7)*		
Male	102(53.1)	61(46.2)	*163(50.3)*	*0.289*	0(0.0)
					
**Disease stage**					
I	26(13.7)	18(13.8)	*44(13.8)*		
II	84(44.2)	50(38.5)	*134(41.9)*		
III	58(30.5)	37(28.5)	*95(29.7)*		
IV	22(11.6)	25(19.2)	*47(14.7)*	*0.284*	4(1.2)
					
**Differentiation grade**					
High-moderate	155(80.7)	95(74.2)	*250(78.1)*		
Poor	37(19.3)	33(25.8)	*70(21.9)*	*0.168*	4(1.2)
					
**Vascular invasion**					
No invasion	164(88.6)	106(86.2)	*270(87.7)*		
Invasion	21(11.4)	17(13.8)	*38(12.3)*	*0.519*	16(4.9)
					
**Mucinous or non-mucinous**					
Non mucinous tumour	161(87.0)	106(86.2)	*267(86.7)*		
Mucinous tumour	24(13.0)	17(13.8)	*41(13.3)*	*0.830*	16(4.9)
					
**Colon or rectal cancer**					
Colon cancer	115(59.6)	102(77.9)	*217(67.0)*		
Rectal cancer	78(40.4)	29(22.1)	*107(33.0)*	*0.001*	0(0)
					
**Colon left or right ex ca recti**					
Right colon	58(50.4)	64(63.4)	*122(56.2)*		
Left colon	57(49.6)	37(36.6)	*94(43.5)*	*0.056*	1(0.5)
					
**CEA serum**					
< 2,67 μg/L	101(52.6)	57(43.2)	*158(48.8)*		
≥ 2,67 μg/L	91(47.4)	75(56.8)	*166(51.2)*	*0.095*	0(0)
					
**Preop radiation therapy**					
Received	129(66.8)	110(84.0)	*239(73.8)*		
Not received	64(33.2)	21(16.0)	*85(26.2)*	*0.001*	0(0)
					
**TATI expression in tissue**					
< 50%	145(81.0)	87(73.7)	*232(78.1)*		
≥ 50%	34(19.0)	31(26.3)	*65(21.9)*	*0.138*	27(8.3)

### Prognostic value of s-TATI and correlation to distant metastases

In ROC analysis the AUC values of both s-CEA and s-TATI ROC correlated significantly with OS (s-TATI; *P *< 0.001, s-CEA; *P *< 0.001) (Figure 1A). This was also the case in curatively treated patients (s-TATI; *P *< 0.001, s-CEA; *P *= 0.002) (Figure 1B). The difference in AUC values between s-TATI and s-CEA was significant in curatively treated patients (*P *= 0.0441) but not in the whole cohort (*P *= 0.346) (Figure 1B).

**Figure 3:**
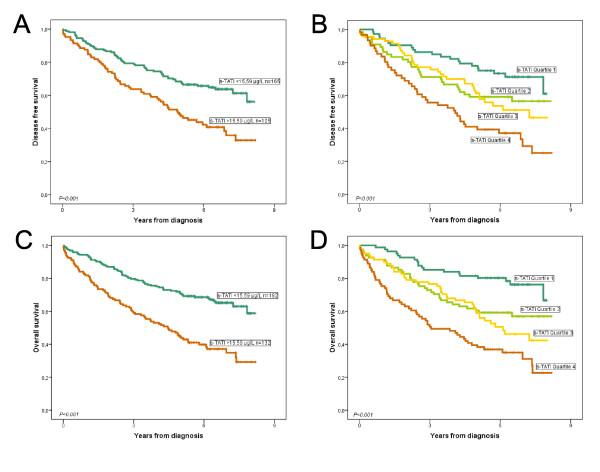
**Prognostic value of s-TATI in all patients**. Kaplan Meier estimates of disease free survival and overall survival in strata of s-TATI levels according to the cut-off derived from the ROC analysis (A, C) and quartiles (B, D).

Analysis of DFS and OS was performed both according to the dichotomized cut-off and according to s-TATI quartiles. Both approaches revealed a significant association (*P *< 0.001) between high concentrations of s-TATI and a shorter DFS and OS (Figure 3). Cox univariate analysis confirmed the association between s-TATI and DFS (HR = 2.00; 95% CI 1.40-2.84, *P *< 0.001) and OS (HR = 2.40; 95% CI 1.74-3.33, *P *< 0.001) (Table 2) and this association remained significant in multivariate analysis adjusted for age, gender, disease stage, differentiation grade, lymphatic or vascular invasion, s-CEA, and t-TATI (HR = 1.51; 95% CI 1.03-2.22, *P *= 0.034 for DFS and HR = 1.78; 95% CI 1.25-2.53, *P *= 0.001 for OS). Notably, when adjusted for s-TATI, t-TATI was an independent prognostic factor for DFS but not for OS (Table 2). The prognostic value of s-CEA analysed using ROC cut-off was more significant for DFS, but less significant than s-TATI as a predictor of OS (Table 2).

**Table 2:** Cox univariate and multivariate analysis of the relative risks for death and recurrent disease according to TATI serum levels.

	Relative risk for death in all patients (Overall survival)			Relative risk for second cancer, recurrence or death to any cause in curatively treated patients (Disease free survival)		
		Univariate	Multivariate		Univariate	Multivariate
	n*	HR (95% CI)	HR (95% CI)	n	HR (95% CI)	HR (95% CI)
**Age at operation**						
Age <75 years	180	1.0 (ref)	1.0 (ref)	148	1.0 (ref)	1.0 (ref)
Age ≥75 years	129	1.88(1.34-2.62)	2.23(1.55-3.23)	116	2.00(1.39-2.86)	1.94(1.30-2.87)
**Gender**						
Female	161	1.0 (ref)	1.0 (ref)	134	1.0 (ref)	1.0 (ref)
Male	164	0.86(0.62-1.18)	0.82(0.58-1.16)	136	0.84(0.59-1.20)	0.82(0.57-1.19)
**Disease stage**						
Stage I	44	1.0 (ref)	1.0 (ref)	44	1.0 (ref)	1.0 (ref)
Stage II	134	0.95(0.52-1.74)	1.07(0.57-2.01)	132	1.36(0.75-2.45)	1.47(0.79-2.70)
Stage III	96	1.84(1.02-3.34)	1.94(1.04-3.65)	92	2.25(1.25-4.06)	2.27(1.21-4.24)
Stage IV	47	7.62(4.11-14.16)	6.88(3.43-13.82)	0	N/A	N/A
**Differentiation grade**						
High-moderate	251	1.0 (ref)	1.0 (ref)	215	1.0 (ref)	1.0 (ref)
Poor	70	1.55(1.07-2.25)	1.38(0.92-2.07)	55	1.31(0.86-2.00)	1.31(0.85-2.02)
**Vascular invasion**						
No invasion	271	1.0 (ref)	1.0 (ref)	243	1.0 (ref)	1.0 (ref)
Invasion	38	2.24(1.45-3.46)	1.29(0.80-2.08)	21	1.67(0.94-2.98)	1.52(0.83-2.78)
**s-CEA**						
< 2,67 μg/L	159	1.0 (ref)	1.0 (ref)	146	1.0 (ref)	1.0 (ref)
≥2,67 μg/L	165	2.05(1.47-2.87)	1.60(1.11-2.32)	124	1.56(1.10-2.23)	1.59(1.10-2.32)
**s-TATI**						
< 15,59 μg/L	159	1.0 (ref)	1.0 (ref)	165	1.0 (ref)	1.0 (ref)
≥15,59 μg/L	165	2.40(1.74-3.33)	1.78(1.25-2.53)	105	2.00(1.40-2.84)	1.51(1.03-2.22)
**t-TATI**						
≤50% cells	233	1.0 (ref)	1.0 (ref)	196	1.0 (ref)	1.0 (ref)
> 50% cells	65	1.40(0.96-2.05)	1.25(0.85-1.86)	58	1.67(1.12-2.49)	1.56(1.04-2.34)

**Figure 4:**
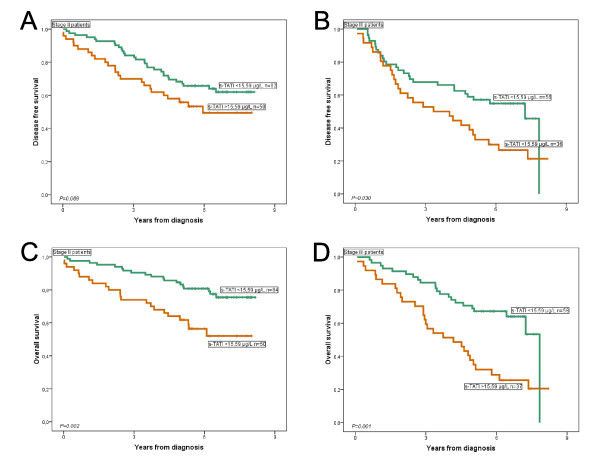
**Prognostic impact of s-TATI in Stage II and Stage III disease**. Kaplan Meier estimates of disease free survival according to high and low s-TATI levels derived from ROC-analysis in Stage II (A) and Stage III patients (B), and overall survival in Stage II (C) and Stage III patients (D), respectively.

The adverse impact of high s-TATI levels on OS was retained also when analyzed separately for cases with stage II and III disease respectively (Figure 4). This was confirmed in Cox univariate analysis (HR = 2.58; 95% CI 1.39-4.79, *P *= 0.003 for Stage II and HR = 2.61; 95% CI 1.49-4.59, *P *= 0.001 for Stage III) and remained significant in multivariate analysis adjusted for age, gender, differentiation grade, lymphatic or vascular invasion, s-CEA and t-TATI (HR = 2.06; 95% CI 1.08-3.93, *P *= 0.028 for Stage II and HR = 2.24; 95% CI 1.19-4.21, *P *= 0.013 for Stage III patients). While a high s-CEA concentration was a significant predictor of reduced OS in patients with Stage II disease in both univariate (HR = 2.17; 95% CI 1.15-4.10, *P *= 0.017) and multivariate analysis (HR = 2.00; 95% CI 1.04-3.87, *P *= 0.039), no significant association was found between s-CEA and OS in patients with Stage III disease (Kaplan Meier curves displayed in Additional file 1, Figure S1).

There was a significant association between high s-TATI levels and a reduced DFS in Stage III patients (Figure 4), also confirmed in Cox univariate analysis (HR = 1.80; 95% CI 1.05-3.07, *P *= 0.033) but not in multivariate analysis (data not shown). In Stage II patients there was no significant association between s-TATI and DFS (Figure 4). S-CEA had no impact on DFS when analyzed separately for patients with Stage II or Stage III disease (Additional file 1, Figure S1). In contrast to previous findings regarding t-TATI [3] there was no relationship between levels of s-TATI and time to liver or lung metastasis (data not shown).

In patients who had not received preoperative RT, s-TATI was an independent prognostic factor (HR = 2.52; 95% CI 1.70-3.72, *P *< 0.001) in univariate analysis and HR = 1.97; 95% CI 1.28-2.99, *P *= 0.002 in multivariate analysis). In patients who had received preoperative RT, s-TATI was prognostic in univariate (HR = 2.27; 95% CI 1.21-4.25, *P *= 0.014) but not multivariate analysis (data not shown).

## Discussion

In this study, we have shown that elevated levels of s-TATI independently predict an adverse outcome in CRC patients and this was also the case in separate analysis of patients with Stage II and III disease.

We have previously shown that high expression of TATI in tumour tissue correlates with an adverse prognosis in two independent cohorts of CRC patients, including the cohort studied here, in which t-TATI was also significantly associated with a reduced time to metachronous liver metastasis [3]. The lack of any association between s-TATI levels and t-TATI is therefore somewhat surprising, given the significant relationship between s-TATI and an adverse prognosis demonstrated in this study. Indeed, s-TATI and t-TATI remained independent prognostic factors when both were included in multivariate analysis. In addition, while s-TATI levels were significantly higher in patients presenting with distant metastasis at diagnosis, we could not confirm any relationship between s-TATI and time to liver or lung metastases.

To our knowledge, the relationship between s-TATI and t-TATI has only been investigated in one previous study on renal cell carcinoma, which also failed to show a significant association between these variables [21]. However, in that study, it was also shown that an impaired renal function could contribute to elevated s-TATI levels in patients with renal cancer. A limitation to this study is the lack of information on renal function for the patients included. The relationship between s-TATI and renal function in CRC patients is an important issue that should be addressed in future studies. In this study, age at diagnosis was significantly associated with high s-TATI levels, which is in line with the study by Solakidi et al. [15]. As high age is associated with a poor DFS and OS in CRC, and also with an impaired renal function, this relationship could to some extent contribute to the adverse prognostic impact of high s-TATI levels. Notably, the independent prognostic value of s-TATI remained significant for both DFS and OS when adjusted for age in the multivariate Cox regression analysis.

Elevation of s-TATI may also be caused by several non-malignant conditions such as severe inflammatory disease [22], pancreatitis and hepatobiliary disease [23], and tissue destruction [24,25]. TATI is produced at high concentration in the pancreas, from which it leaks into serum in pancreatic disease [24]. However, normal s-TATI levels have been found after total pancreatectomy showing that TATI is produced in several tissues [26,27]. Solakidi *et al *suggested that leakage from the tumour cells into the circulation might be the mechanism behind elevated s-TATI levels [15], but the lack of a correlation between s-TATI and t-TATI in this study implies that leakage from the tumour into the circulation is not a major cause of elevated s-TATI levels. Apart from its trypsin inhibiting function, TATI has also been ascribed properties promoting a more malignant phenotype in CRC [4] and in prostate cancer cells [28]. Interestingly, TATI has been shown to stimulate the EGF receptor and thus increase tumour aggressiveness [29]. Therefore, it could well be that t-TATI contributes to the biological aggressiveness of the tumour per se, whereas s-TATI levels reflect a local and/or systemic response to the disease.

Notably, while there was no association between t-TATI and tumour location, s-TATI levels were significantly lower in left-sided tumours compared to right-sided tumours and in rectal cancers compared to colonic cancers, the majority of which had received preoperative RT. None of these associations were seen for s-CEA. The significant association between lower s-TATI levels and RT indicates that treatment might directly affect s-TATI levels. This would be of interest to investigate in future studies, comparing levels of s-TATI before, during and after RT. In this study, s-TATI was an independent prognostic factor in patients not receiving RT, whereas it was only prognostic in univariate analysis in patients treated with RT. As the number of patients that had received RT was relatively small (n = 85) and the number of patients that had not received RT was even smaller (n = 23), no conclusions can be drawn regarding the value of s-TATI as a predictor of response to preoperative RT in rectal cancer patients. This should be investigated in a prospective setting, preferably a randomised trial.

Notably, the significantly lower s-TATI levels in left-sided compared to right-sided colonic tumours indicate that differences in s-TATI levels according to tumour location might also be due to differences in biological characteristics of the tumours, e.g., microsatellite instability (MSI), which is more frequently present in tumours arising in the right colon [30,31]. Information on MSI status was not available for the tumours in this study, but should be considered in future studies.

Earlier studies on s-TATI in colorectal cancer have only concerned its diagnostic value, and for this purpose s-TATI has not been found to provide information additional to that obtained with s-CEA [13,14]. The prognostic value of s-TATI in CRC has not been studied before. In the present study s-TATI was found to be a prognostic biomarker that was independent of conventional clinicopathological factors, and also independent of s-CEA. Furthermore, in most settings, the value of s-TATI was stronger than that of s-CEA.

Individualised treatment is still not available for patients with CRC in the adjuvant setting; currently the TNM stage is used for selection of patients for adjuvant treatment. In stage II patients about 20% will develop cancer recurrence, and there is modest evidence that these patients benefit from adjuvant chemotherapy [32]. Several high risk factors have been suggested for selecting stage II patients for adjuvant chemotherapy, i.e few lymph nodes examined, T4 disease, obstruction and tumour perforation [33]. Stage III CRC patients, on the other hand, have a well documented benefit from adjuvant chemotherapy [34] although it is estimated that up to 80% will relapse despite adjuvant treatment. It is therefore obvious that more accurate prognostic factors are needed when selecting patients for adjuvant treatment in CRC. The data presented here show that s-TATI is an independent prognostic biomarker in the whole cohort and in separate analysis of patients with Stage II and III disease.

## Conclusions

In summary, we have demonstrated that TATI in serum, although not associated with its tumour-specific expression in tissue, is an independent predictor of a poor outcome in CRC patients. As this was evident in separate analysis of Stage II and Stage III patients, the prognostic value of s-TATI should be studied further to clarify whether it can be used as a biomarker for decision making regarding the benefit from adjuvant chemotherapy in patients with Stage II disease and the intensity of adjuvant chemotherapy needed in patients with Stage III disease.

## Competing interests

The authors declare that they have no competing interests.

## Authors' contributions

AG participated in the collection of data, performed statistical analyses and drafted the manuscript. BN assisted with the serum analyses and revision of the manuscript. EN assisted with the serum analyses. KH performed the serum analyses and revised the manuscript. UHS participated in the design of the study and helped with revision of the manuscript. AB participated in the design of the study and helped with the revision of the manuscript. HB included the patients into the study, collected data, drafted the manuscript and participated in the statistical analysis. KJ participated in the conception and design of the study, statistical analysis, drafted and revised the manuscript. All authors read and approved the final manuscript.

## Pre-publication history

The pre-publication history for this paper can be accessed here:

http://www.biomedcentral.com/1471-2407/10/498/prepub

## Supplementary Material

Additional file 1 **Prognostic impact of s-CEA in Stage II and Stage III disease**. Kaplan Meier estimates of disease free survival according to high and low s-CEA levels derived from ROC-analysis in Stage II (A) and Stage III patients (B), and overall survival in Stage II (C) and Stage III patients (D), respectively. Click here for file

## References

[B1] Parkin DM Bray F Ferlay J Pisani P Global cancer statistics, 2002 CA Cancer J Clin 2005 55 2 74 108 10.3322/canjclin.55.2.74 15761078

[B2] Iqbal S Stoehlmacher J Lenz HJ Tailored chemotherapy for colorectal cancer: a new approach to therapy Cancer Invest 2004 22 5 762 773 10.1081/CNV-200032774 15581057

[B3] Gaber A Johansson M Stenman UH Hotakainen K Ponten F Glimelius B Bjartell A Jirstrom K Birgisson H High expression of tumour-associated trypsin inhibitor correlates with liver metastasis and poor prognosis in colorectal cancer British journal of cancer 2009 100 10 1540 1548 10.1038/sj.bjc.6605047 19384300 PMC2696764

[B4] Gouyer V Fontaine D Dumont P de Wever O Fontayne-Devaud H Leteurtre E Truant S Delacour D Drobecq H Kerckaert JP Autocrine induction of invasion and metastasis by tumor-associated trypsin inhibitor in human colon cancer cells Oncogene 2008 27 29 4024 4033 10.1038/onc.2008.42 18317448

[B5] Venesmaa P Lehtovirta P Stenman UH Leminen A Forss M Ylikorkala O Tumour-associated trypsin inhibitor (TATI): comparison with CA125 as a preoperative prognostic indicator in advanced ovarian cancer British journal of cancer 1994 70 6 1188 1190 7981075 10.1038/bjc.1994.471 PMC2033663

[B6] Paju A Vartiainen J Haglund C Itkonen O von Boguslawski K Leminen A Wahlstrom T Stenman UH Expression of trypsinogen-1, trypsinogen-2, and tumor-associated trypsin inhibitor in ovarian cancer: prognostic study on tissue and serum Clin Cancer Res 2004 10 14 4761 4768 10.1158/1078-0432.CCR-0204-03 15269150

[B7] Pectasides D Bafaloucos D Antoniou F Gogou L Economides N Varthalitis J Dimitriades M Kosmidis P Athanassiou A TPA, TATI, CEA, AFP, beta-HCG, PSA, SCC, and CA 19-9 for monitoring transitional cell carcinoma of the bladder Am J Clin Oncol 1996 19 3 271 277 10.1097/00000421-199606000-00013 8638540

[B8] Kelloniemi E Rintala E Finne P Stenman UH Tumor-associated trypsin inhibitor as a prognostic factor during follow-up of bladder cancer Urology 2003 62 2 249 253 10.1016/S0090-4295(03)00329-7 12893328

[B9] Paju A Jacobsen J Rasmuson T Stenman UH Ljungberg B Tumor associated trypsin inhibitor as a prognostic factor in renal cell carcinoma The Journal of urology 2001 165 3 959 962 10.1016/S0022-5347(05)66584-6 11176522

[B10] Meria P Toubert ME Cussenot O Bassi S Janssen T Desgrandchamps F Cortesse A Schlageter MH Teillac P Le Duc A Tumour-associated trypsin inhibitor and renal cell carcinoma European urology 1995 27 3 223 226 7601186 10.1159/000475165

[B11] Taccone W Mazzon W Belli M Evaluation of TATI and other markers in solid tumors Scandinavian journal of clinical and laboratory investigation 1991 207 25 32 10.3109/00365519109104622 1780686

[B12] Satake K Inui A Sogabe T Yoshii Y Nakata B Tanaka H Chung YS Hiura A Umeyama K The measurement of serum immunoreactive pancreatic secretory trypsin inhibitor in gastrointestinal cancer and pancreatic disease Int J Pancreatol 1988 3 5 323 331 2459273 10.1007/BF02788466

[B13] Pasanen P Eskelinen M Kulju A Penttila I Janatuinen E Alhava E Tumour-associated trypsin inhibitor (TATI) in patients with colorectal cancer: a comparison with CEA, CA 50 and CA 242 Scand J Clin Lab Invest 1995 55 2 119 124 10.3109/00365519509089603 7667604

[B14] Catarino M Conde R Tumor-associated trypsin inhibitor (TATI) in patients with colorectal carcinoma A critical comparison with CEA. Scandinavian journal of clinical and laboratory investigation 1991 207 43 46 10.3109/00365519109104625 1780689

[B15] Solakidi S Dessypris A Stathopoulos GP Androulakis G Sekeris CE Tumour-associated trypsin inhibitor, carcinoembryonic antigen and acute-phase reactant proteins CRP and alpha1-antitrypsin in patients with gastrointestinal malignancies Clin Biochem 2004 37 1 56 60 10.1016/j.clinbiochem.2003.09.002 14675563

[B16] Stenman UH Koivunen E Itkonen O Biology and function of tumor-associated trypsin inhibitor, TATI Scandinavian journal of clinical and laboratory investigation 1991 207 5 9 10.3109/00365519109104618 1780691

[B17] Matsuda K Ogawa M Shibata T Nishibe S Miyauchi K Matsuda Y Mori T Postoperative elevation of serum pancreatic secretory trypsin inhibitor Am J Gastroenterol 1985 80 9 694 698 2412437

[B18] Eddeland A Ohlsson K A radioimmunoassay for measurement of human pancreatic secretory trypsin inhibitor in different body fluids Hoppe-Seyler's Zeitschrift fur physiologische Chemie 1978 359 6 671 675 669578 10.1515/bchm.1978.359.1.671

[B19] Ogawa M Pancreatic secretory trypsin inhibitor as an acute phase reactant Clin Biochem 1988 21 1 19 25 10.1016/S0009-9120(88)80107-3 2449986

[B20] Punt CJ Köhne Claus-Henning Hohenberger Peter Labianca Roberto Hans Schmoll J Påhlman Lars Alberto Sobrero J-YD Endpoints in Adjuvant Treatment Trials: A Systematic Review of the Literature in Colon Cancer and Proposed Definitions for Future Trials J Natl Cancer Inst 2007 99 998 1003 10.1093/jnci/djm024 17596575

[B21] Lukkonen A Lintula S von Boguslawski K Carpen O Ljungberg B Landberg G Stenman UH Tumor-associated trypsin inhibitor in normal and malignant renal tissue and in serum of renal-cell carcinoma patients Int J Cancer 1999 83 4 486 490 10.1002/(SICI)1097-0215(19991112)83:4<486::AID-IJC9>3.0.CO;2-O 10508484

[B22] Huhtala ML Kahanpaa K Seppala M Halila H Stenman UH Excretion of a tumor-associated trypsin inhibitor (TATI) in urine of patients with gynecological malignancy Int J Cancer 1983 31 6 711 714 10.1002/ijc.2910310606 6190763

[B23] Haglund C Huhtala ML Halila H Nordling S Roberts PJ Scheinin TM Stenman UH Tumour-associated trypsin inhibitor, TATI, in patients with pancreatic cancer, pancreatitis and benign biliary diseases British journal of cancer 1986 54 2 297 303 3741764 10.1038/bjc.1986.176 PMC2001515

[B24] Hedstrom J Haglund C Leinonen J Nordling S Stenman UH Trypsinogen-1, -2 and tumour-associated trypsin-inhibitor in bile and biliary tract tissues from patients with biliary tract diseases and pancreatic carcinomas Scand J Clin Lab Invest 2001 61 2 111 118 10.1080/00365510151097584 11347977

[B25] Ogawa M Matsuda K Shibata T Matsuda Y Ukai T Ohta M Mori T Elevation of serum pancreatic secretory trypsin inhibitor (PSTI) in patients with serious injury Res Commun Chem Pathol Pharmacol 1985 50 2 259 266 2417295

[B26] Lasson A Borgstrom A Ohlsson K Serum levels of immunoreactive PSTI in acute abdominal disorders, with special reference to a possible extrapancreatic PSTI production Clin Chim Acta 1986 161 1 37 46 10.1016/0009-8981(86)90261-5 2434267

[B27] Halila H Huhtala ML Schroder T Kiviluoto T Stenman UH Pancreatic secretory trypsin inhibitor-like immunoreactivity in pancreatectomized patients Clin Chim Acta 1985 153 3 209 216 10.1016/0009-8981(85)90354-7 3935345

[B28] Tomlins SA Rhodes DR Yu J Varambally S Mehra R Perner S Demichelis F Helgeson BE Laxman B Morris DS The role of SPINK1 in ETS rearrangement-negative prostate cancers Cancer cell 2008 13 6 519 528 10.1016/j.ccr.2008.04.016 18538735 PMC2732022

[B29] Ozaki N Ohmuraya M Hirota M Ida S Wang J Takamori H Higashiyama S Baba H Yamamura K Serine protease inhibitor Kazal type 1 promotes proliferation of pancreatic cancer cells through the epidermal growth factor receptor Mol Cancer Res 2009 7 9 1572 1581 10.1158/1541-7786.MCR-08-0567 19737965

[B30] Miyakura Y Sugano K Konishi F Ichikawa A Maekawa M Shitoh K Igarashi S Kotake K Koyama Y Nagai H Extensive methylation of hMLH1 promoter region predominates in proximal colon cancer with microsatellite instability Gastroenterology 2001 121 6 1300 1309 10.1053/gast.2001.29616 11729109

[B31] Hawkins N Norrie M Cheong K Mokany E Ku SL Meagher A O'Connor T Ward R CpG island methylation in sporadic colorectal cancers and its relationship to microsatellite instability Gastroenterology 2002 122 5 1376 1387 10.1053/gast.2002.32997 11984524

[B32] Quasar Collaborative G Gray R Barnwell J McConkey C Hills RK Williams NS Kerr DJ Adjuvant chemotherapy versus observation in patients with colorectal cancer: a randomised study Lancet 2007 370 9604 2020 2029 10.1016/S0140-6736(07)61866-2 18083404

[B33] Figueredo A Coombes ME Mukherjee S Adjuvant therapy for completely resected stage II colon cancer Cochrane Database Syst Rev 2008 3 CD005390 18646127 10.1002/14651858.CD005390.pub2 PMC8885310

[B34] Sargent D Sobrero A Grothey A O'Connell MJ Buyse M Andre T Zheng Y Green E Labianca R O'Callaghan C Evidence for cure by adjuvant therapy in colon cancer: observations based on individual patient data from 20,898 patients on 18 randomized trials J Clin Oncol 2009 27 6 872 877 10.1200/JCO.2008.19.5362 19124803 PMC2738431

